# Chest CT for suspected pulmonary complications of oncologic therapies: how I review and report

**DOI:** 10.1186/s40644-016-0066-4

**Published:** 2016-04-11

**Authors:** Stefan Diederich

**Affiliations:** Department of Diagnostic and Interventional Radiology, Marien Hospital, Academic Teaching Hospital, Rochusstr. 2, D- 40479 Düsseldorf, Germany

**Keywords:** Lung, Radiation pneumonitis, Drug toxicity, Infection, Cryptogenic organizing pneumonia, Oedema, Computed tomography, Image interpretation, Image display

## Abstract

In cancer patient during or following oncologic therapies with respiratory symptoms and pulmonary pathology at chest CT the differential diagnosis includes infection, therapy-induced disease and tumour progression.

Although CT morphology may be typical or even pathognomonic in some conditions the diagnosis is usually made by a synopsis of imaging, clinical and laboratory features.

Close communication with referring colleagues and a good knowledge of potential side effects of therapeutic concepts, their time course and CT morphology is crucial in the differential diagnosis.

This review describes a personal approach to the radiological diagnosis of therapy-induced pulmonary abnormalities in cancer patients.

## Background

### Why imaging for pulmonary complications of oncologic therapy?

In patients during or following therapy for cancer pulmonary symptoms are common. These may, obviously, be due to a variety of causes such as progression of malignancy involving the chest as well as infectious or non-infectious complications of surgery, radiation or medical therapy. In addition, in cancer patients disease unrelated to malignancy may occur, although the risk for some of these conditions may be increased due to the disease or its therapy.

The different potential causes of chest symptoms may require completely different therapeutic approaches. Escalation or change of chemotherapy or molecular therapy may be required in progressive disease but may have catastrophic results in pulmonary toxicity of these drugs. Steroid therapy is usually indicated in non-infectious inflammatory complications but may aggravate pulmonary infection. Antimicrobial treatment is obviously useful in infectious complications but its toxicity particularly of antifungal therapy may be detrimental in cases of non-infectious disease. Unnecessary discontinuation of an effective drug will obviously result in a negative effect for the patient.

Furthermore, in cancer patients pulmonary disease may have a more aggressive and potentially lethal course than in otherwise healthy patients.

For all these reasons it is mandatory to establish the cause of the pulmonary symptoms fast and reliably.

The clinical signs and symptoms of pulmonary disease such as cough, dyspnoea, hypoxia and signs of inflammation are usually unspecific and do not reliably allow differentiation between the different conditions.

Laboratory test alone are usually not specific enough for patient management. Therefore, imaging is crucial in these circumstances and has a major impact on therapeutic decisions.

This review describes a personal approach to the radiological diagnosis of therapy-induced pulmonary abnormalities in cancer patients. It does not claim to cover all aspects of the problem and specifically does not include post-surgical changes.

## Risk considerations: assessing pre-test probability of different pulmonary complications

As almost all imaging findings in pulmonary complications are not specific enough to decide on the significant therapeutic consequences on the basis of imaging results alone it is of paramount importance to use a synopsis of clinical laboratory and imaging data to make the diagnosis.

### Pulmonary disease in oncologic therapy

#### Radiation pneumonitis

Radiation of pulmonary parenchyma exceeding a dose of 30–40 Gray (Gy) usually leads to radiation pneumonitis which may be clinically occult but may also present with symptoms such as unproductive cough, dyspnoea and clinical and laboratory signs of inflammation.

Radiation pneumonitis follows a rather typical time course: 6–10 weeks after the radiation dose threshold has been exceeded ground glass is observed which then increases in density to present as consolidation. After some months fibrosis occurs with a decrease in volume of the involved lung area and signs such as traction bronchiectasis and displacement such as interlobar fissures, vessels and bronchi.

Radiation pneumonitis almost exclusively involves the lung area that was affected by radiation dose above the threshold and is not limited by anatomical borders such as interlobar fissures.

**Fig. 1:**
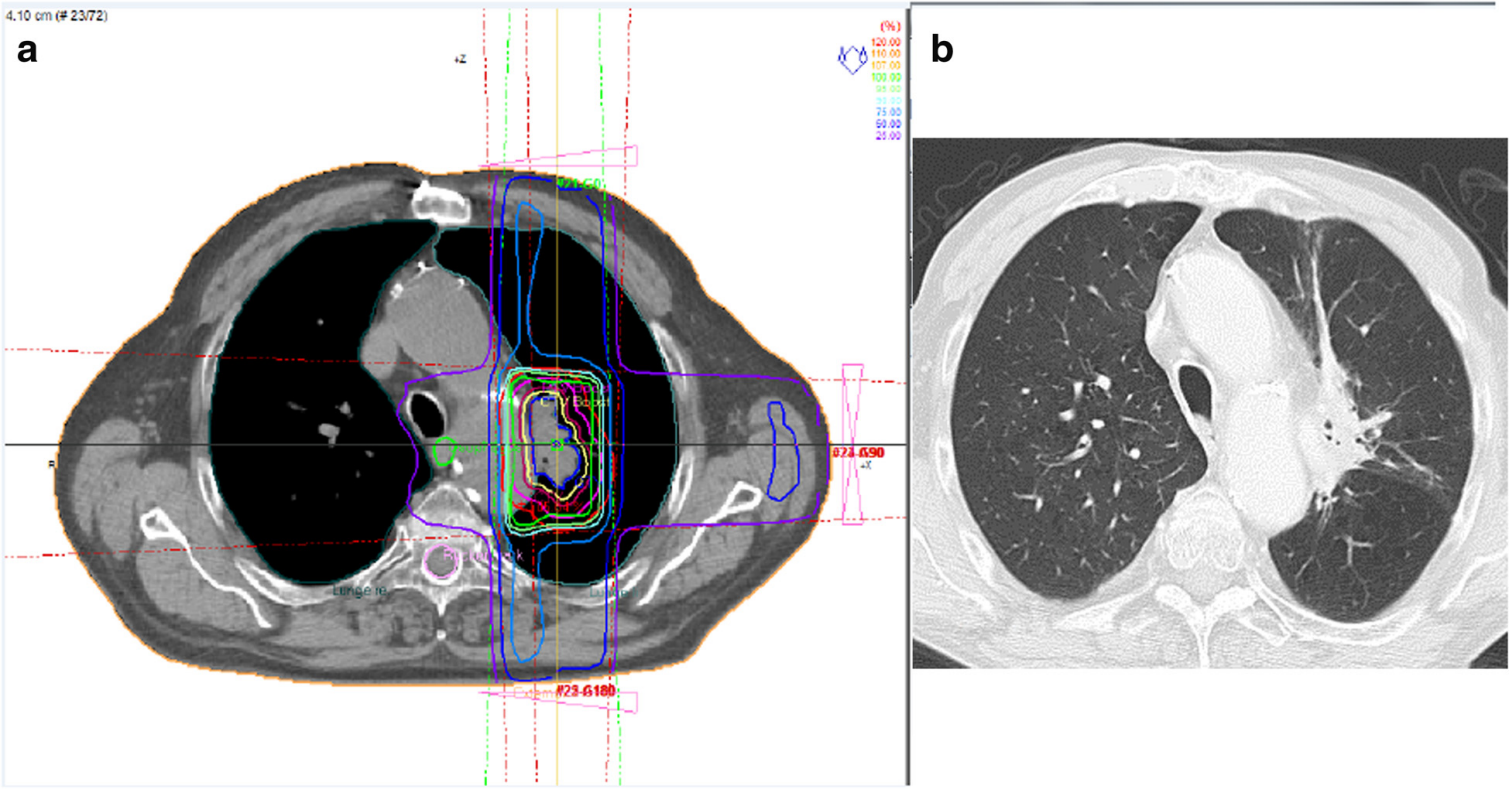
Radiation pneumonitis. Patient with NSCLC (non-small cell lung cancer) treated with radiation. **a** Dose distribution at radiation planning simulation. **b** Chest CT at lung window with consolidation. Note the tongue-like area of consolidation in the anterior segment of the left upper lobe reflecting the area with > 75 % of the total dose at the dose distribution plan

It is relatively easy to make a diagnosis of radiation pneumonitis in simple radiation ports as there is usually a sharp border between involved and normal lung following the borders of the radiation port [1,2]. If, however, more modern radiotherapy techniques are used (e.g. intensity-modulated radiation therapy (iMRT), gamma-knife, cyber-knife) it may be impossible to establish the diagnosis unless the dose distribution is known (Fig. 1). Ideally, radiation planning data should be available to the diagnostic radiologist in these patients.

### Drug-induced pulmonary toxicity

A large variety of drugs is used in modern systemic therapy of malignancy and usually combinations of two or more drugs are administered in order to increase the efficacy without increasing toxicity. Classical cytotoxic chemotherapy, leading to cell tumour necrosis may be combined with molecular therapy that blocks cell metabolism, blood supply and other cellular functions without actually destroying the tumour cells. Most recently immune therapy has been introduced in which the host’s immune response to the malignant cells is enhanced [3].

Many of these agents have side effects that may manifest in the lung. Due to the fact that there are innumerable combinations of different drugs with different doses it is very difficult to predict potential toxic effects in the lung. Furthermore, the spectrum of drug-induced pulmonary disease is not unique but represents a spectrum of diseases which also occurs unrelated to drug toxicity.

**Fig. 2:**
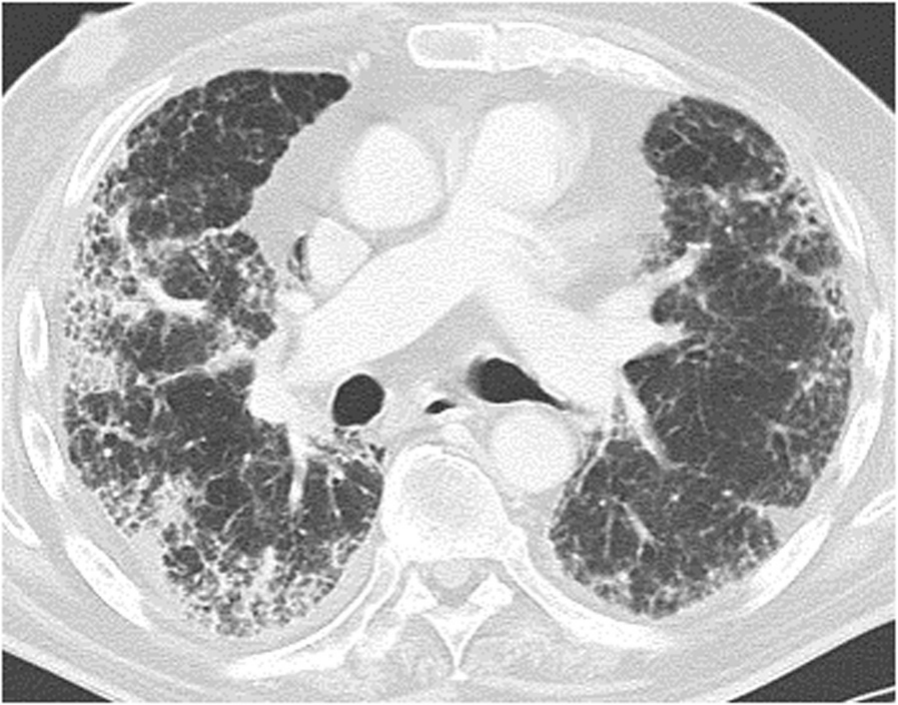
Drug-induced lung disease with a NSIP pattern (non-specific interstitial pneumonia). Patient with chemotherapy for bladder cancer. Chest CT at the level of the right pulmonary artery at lung window. Diffuse bilateral peripheral reticular pattern, ground glass and some consolidation

It includes hypersensitivity pneumonitis, interstitial pneumonitis with a pattern of non-specific interstitial pneumonia (NSIP) (Fig. 2), cryptogenic organizing pneumonia (COP), pulmonary haemorrhage, pulmonary oedema, bronchiolitis, vasculitis and many others.

Finally, one drug may cause several different patterns of pulmonary toxicity. Therefore, it is almost impossible to know all possible patterns of drug-induced lung disease even in the commonest therapeutic regimens. There is a very helpful freely available website which lists all potential effects of a large variety of drugs (not only in oncology) which is regularly updated (http://www.pneumotox.com). The website does not present radiological images but lists potential patterns.

### Infection: predisposing factors for infection with different organisms

Obviously, immunosuppression due to malignancy (e.g. lymphoma, leukaemia) or its therapy (particularly chemotherapy, molecular therapy) often increases the risk of pulmonary infection.

Furthermore, depending on the affected cell line and other factors the risk for infection with specific organisms differs. If these conditions are taken into account it is possible to estimate whether a patient is more likely to develop bacterial, fungal, viral infection.

**Fig. 3:**
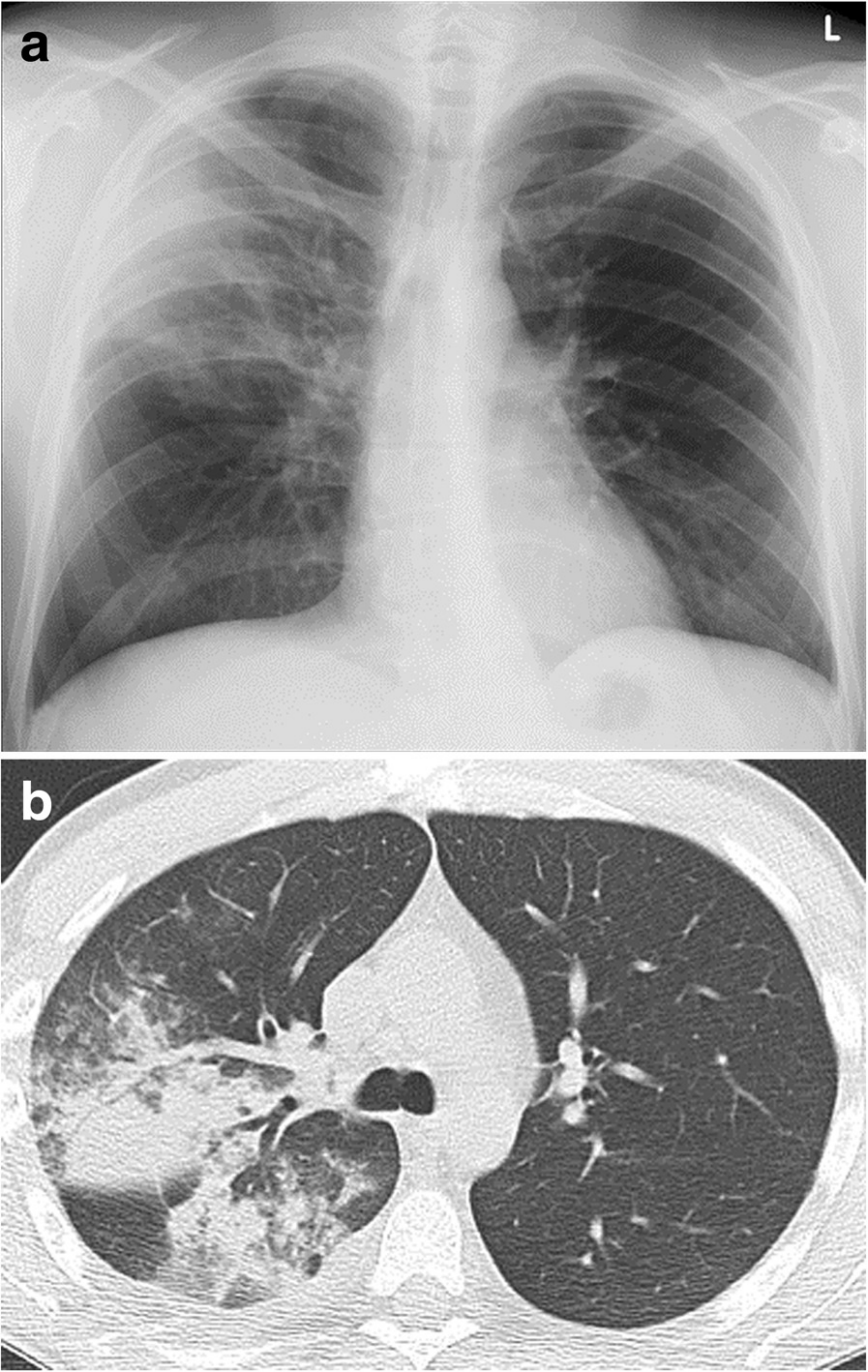
Bacterial pneumonia. Patient with chemotherapy for Burkitt-Lymphoma. **a** Chest radiograph p.a.: Consolidation projected over the right lateral upper lung zone. **b** Chest CT at lung window: heterogenous consolidation in the posterior segment of the right upper lobe and the apical segment of the right lower lobe

**Fig. 4:**
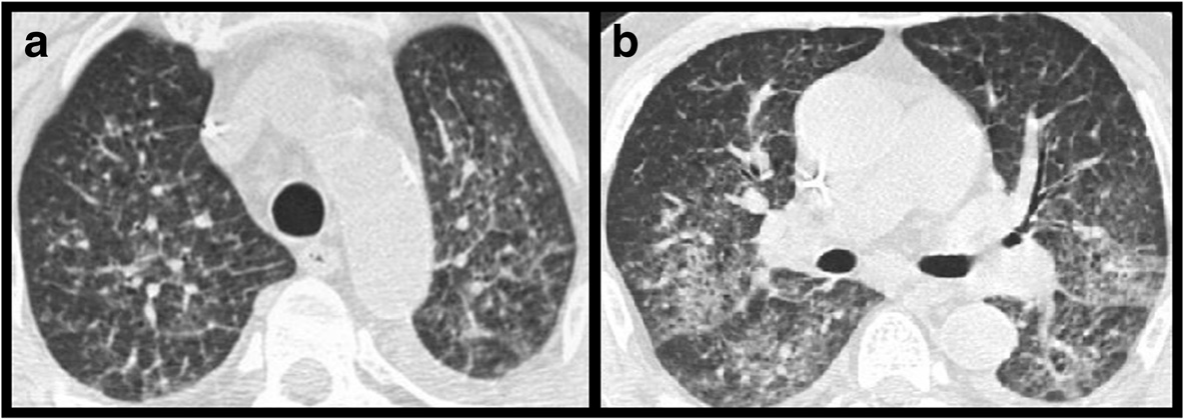
Viral pneumonia from cytomegaly virus. Patient with chemotherapy for Non-Hodgkin Lymphoma. **a**, **b** Chest CT at the level of the aortic arch (**a**) and the pulmonary trunk (**b**) at lung windows. Bilateral reticular-nodular interstitial pattern, consolidation and ground glass

**Fig. 5:**
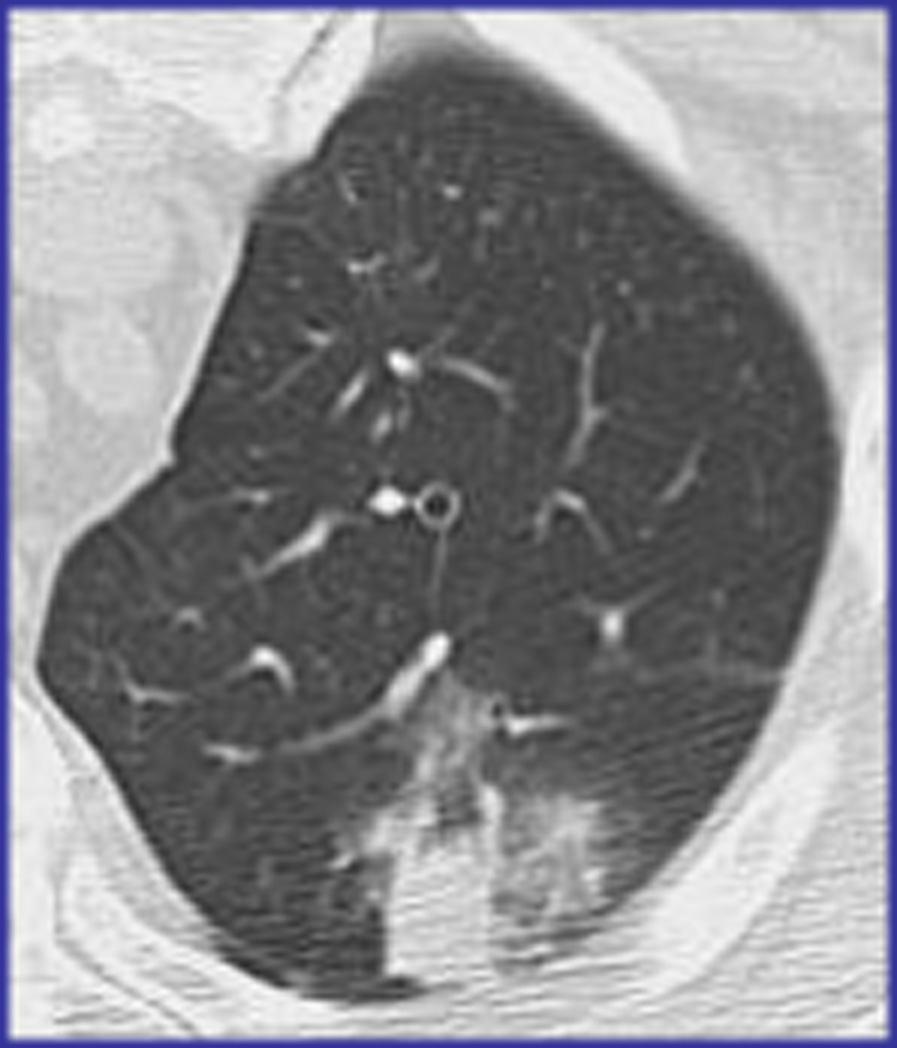
Angioinvasive pulmonary aspergillosis. Patient with chemotherapy for acute myeloid leukaemia. Chest CT at the level of the apical segment of the left upper lobe at lung window: Focal area of consolidation surrounded by a “halo” of ground glass

**Fig. 6:**
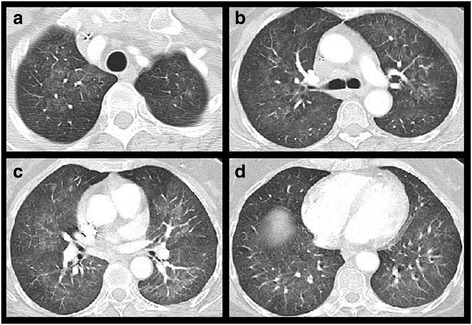
Pneumocystis jirovecii pneumonia. Patient with adjuvant chemotherapy for breast cancer. Chest CT at the level of the lung apex (**a**), tracheal bifurcation (**b**), apical segments of the lower lobes (**c**) and the dome of the right diaphragm (**d**) at lung windows: Diffuse, symmetrical, bilateral ground glass sparing the lung periphery both in the axial plane as well as lung apices and bases

There is however, an overlap and a combination of infection with different organisms at the same time is not uncommon (Table 1) (Figs. 3, 4, 5 and 6).

**Table 1:** Risk factors predisposing to infection with different organisms

Involved cell line	Granulocytes, Monocytes	B-Lymphocytes	T-Lymphocytes
Immune defect	Unspecific cellular defense	Antibody-deficiency	Specific cellular defense
Disease	Solid tumours myeloid leukaemia short term (<5 d) neutropenia	B-cell-lymphoma, B-ALL, lymphotoxic therapy (steroids)	T-cell-lymphoma, T-ALL, lymphotoxic therapy continuous immunosuppression, allogenic stem cell transplant, organ transplant, T-cell-specific drugs (Alemzutumab, Antithymocyte globulin)
Organisms	Bacteria (gram+, gram-) in neutropenia > 10 d also fungi (lung: Aspergillus, liver/spleen: Candida, pleura/paranasal sinus: Mucormyces)	Bacteria (gram +, gram-)	Pneumocystis jirovecii (PCP), viruses, fungi (Toxoplasma, Listeria, Coccidiomyces)
		Pneumocystis jirovecii (PCP)	

Adapted from: Diederich S and Giagounidis A (2014) Therapy-associated changes of the lung and pleura in cancer patients. RadiologieUp2date 14: 331-346

### Other disease with increased risk in cancer patients

**Cryptogenic organizing pneumonia (COP)** manifests as solitary or multiple peripheral areas of consolidation, pulmonary nodules or masses, peribronchovascular consolidation or other morphology.

It may occur secondary to infection, therapy with different drugs, collagen vascular diseases or with no known cause (idiopathic COP).

In cancer patients it may be a manifestation of drug-induced lung disease. It is also associated with radiation therapy. Other than radiation pneumonitis the lung abnormalities are not confined to the radiation port and the time course is variable with COP occurring weeks or months after radiation therapy [4].

**Deep venous thrombosis** (DVT) leading to **pulmonary embolism** (PE) is more common in cancer patients than in patients with no malignancy. It may be due to the cancer (e.g. particularly increased risk in pancreatic cancer) or its therapy (e.g. antihormonal therapy in hormone receptor-positive breast cancer) [5].

**Fig. 7:**
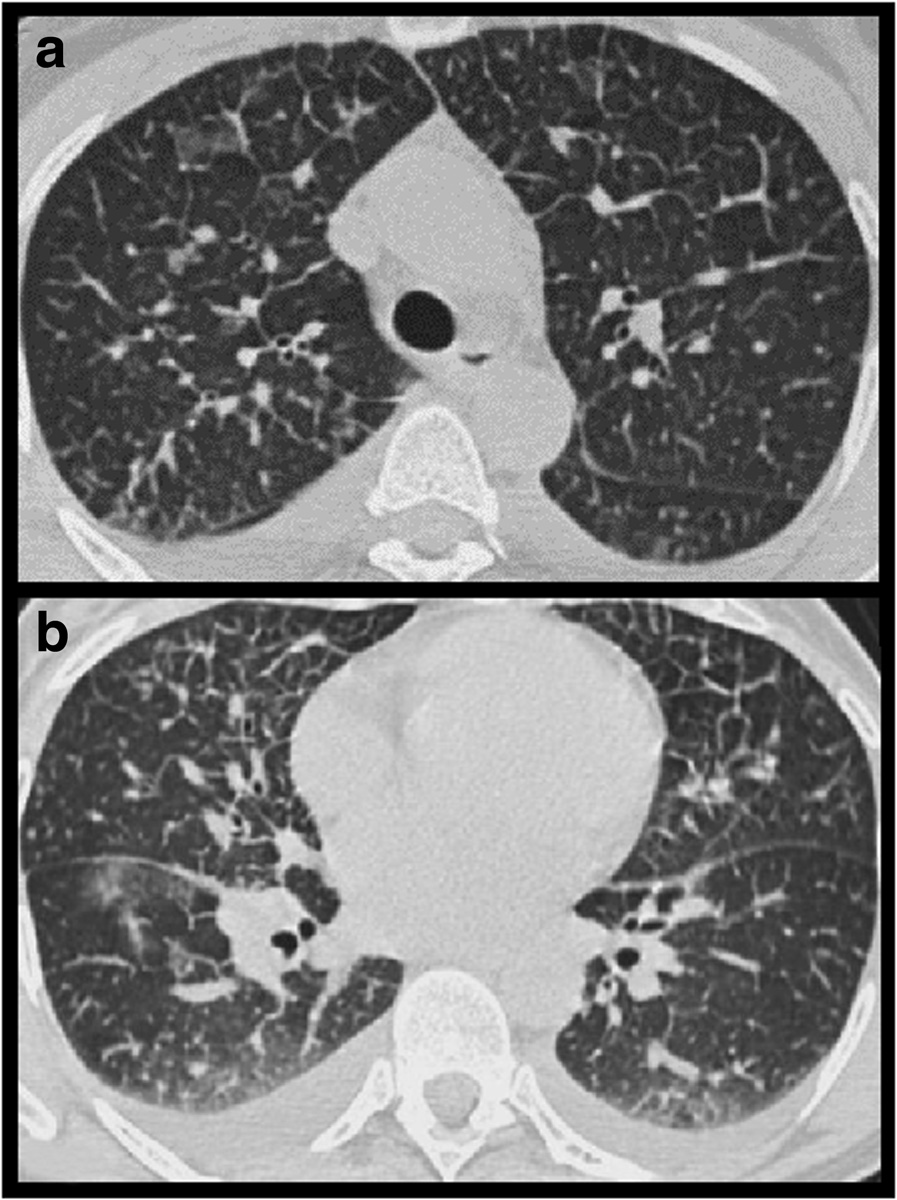
Pulmonary interstitial and alveolar oedema. Patient with adjuvant chemotherapy for breast cancer. **a**, **b** chest CT at the level of the aortic arch (**a**) and the right lower pulmonary vein (**b**) at lung windows: Diffuse bilateral reticular pattern due to interlobular septal thickening, some mild ground glass, mild thickening of bronchovascular bundles and bilateral pleura l effusions, right larger than left

**Pulmonary oedema** is not uncommon in patients undergoing chemotherapy as many regimens include large amounts of fluid in order to decrease the local toxicity of the drugs. Particularly in patients with renal insufficiency this may lead to a fluid overload resulting in pulmonary interstitial or even intraalveolar oedema (Fig. 7).

### Progression of malignancy in the lung

In cancer patients pulmonary complications of the therapy, obviously need to be differentiated from pulmonary manifestations or progression of malignancy.

Lymphangitic carcinomatosis (LAC) usually presents with dyspnoea, unproductive cough and may be associated with signs of inflammation. It, typically, manifests radiologically as reticular and linear pattern due to smooth or nodular thickening of interlobar and interlobular septae and thickening of bronchovascular bundles. It may be associated with pleural effusion with or without nodular pleural thickening, lymphadenopathy and other signs of tumour progression.

**Malignant lymphoma** may involve the lung either with a solitary lesion (extranodal manifestations) or diffuse lung involvement (stage IV disease). Pulmonary lymphoma may present as well- or ill-defined solitary or multiple nodules, areas of consolidation mimicking pneumonia or interstitial pattern (thickened septae and bronchovascular bundles).

**Fig. 8:**
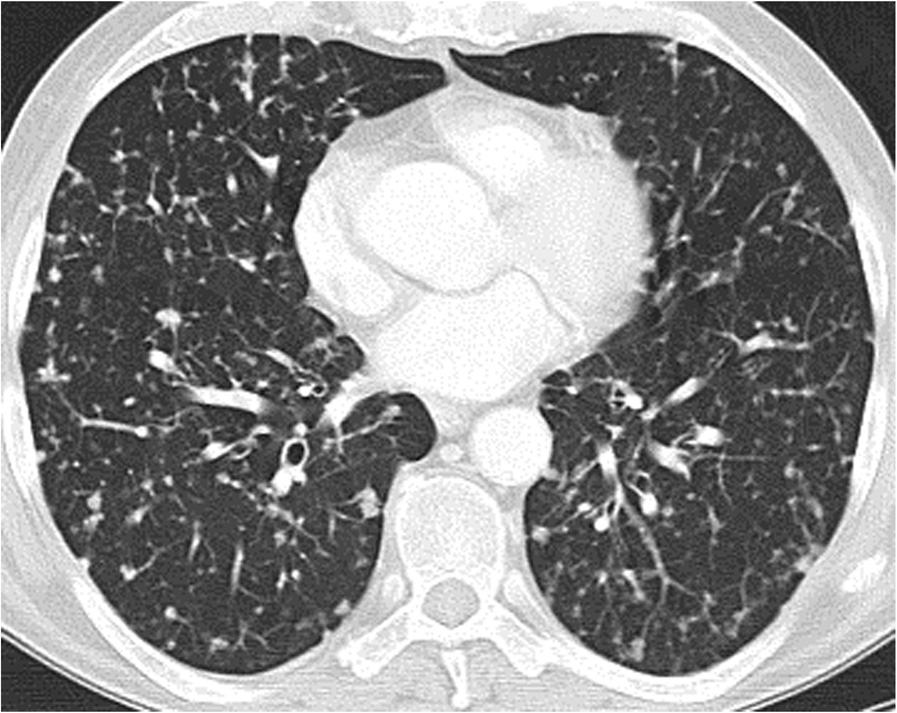
Multiple pulmonary metastases. Patient with sigmoid carcinoma. Chest CT at the level of the right inferior pulmonary vein at lung window: Multiple small pulmonary nodules with different sizes and random distribution in all lung lobes

**Pulmonary metastases** usually present as multiple, well-defined solid nodules with a predominance in the lower lung zones and the lung periphery. Ill-defined, solitary or central nodules are less common. Typically, metastases exhibit a nodular pattern with different-size nodules (Fig. 8).

### Imaging techniques

#### Chest radiography

Conventional radiography should be performed if at all possible erect in two views (p.a. and lateral). A high quality chest radiograph is appropriate to confirm or exclude pulmonary disease in most clinical settings.

If the patient’s clinical situation allows only a.p. supine chest radiography this may be insufficient because of its limitations in both sensitivity and specificity for pulmonary disease and chest CT may be required.

## Is there a role for chest CT in patients with a normal chest radiograph?

There are clinical settings in which chest CT is indicated even in the presence of a normal high quality chest radiograph in two views.

In patients with **severe neutropenia** (<1000 neutrophil granulocytes/μl) the usual response to organisms causing pulmonary infection of neutrophils migrating to the site of infection causing an inflammatory infiltrate composed of fluid, neutrophils, macrophages and lymphocytes may not be possible due to a lack of the cellular elements.

In this situation imaging is not aimed at demonstrating the host’s response to the infection (i.e. inflammatory infiltration presenting as consolidation or ground glass opacity) but rather the local effects of the offending organism itself such as local haemorrhage or vascular occlusion due to invasion by the organism.

Patients with prolonged (>5 days) neutropenia are particularly susceptible to **fungal infection** (aspergillus, candida etc.) and antifungal therapy is associated with significant toxicity. Therefore, it is advocated to perform chest CT even in the presence of a normal chest radiograph, particularly if empirical antibacterial therapy does not result in a resolution of the symptoms (Fig. 4).

Another specific situation in which chest radiography may be insufficient to demonstrate significant pulmonary disease is **pneumocystis jirovecii pneumonia (PCP)**. In the early stages of the infection this organism causes diffuse ground glass which may be impossible to detect even at high quality erect two view chest radiography. The only but obviously unspecific sign at chest radiography may be decreased depth of inspiration compared to previous chest films due to a decrease in elasticity of the pulmonary parenchyma.

Therefore, in patients with suspected PCP chest CT may be helpful to demonstrate the typical pattern of ground glass opacity sparing the lung periphery with no associated lymphadenopathy or pleural effusion (Fig. 6).

### CT technique

#### Intravenous contrast medium

If the detection and/or classification of pulmonary pathology is the sole indication for imaging unenhanced CT is usually sufficient.

Intravenous administration of contrast medium with a delay sufficient to allow enhancement of soft tissues (40–70 s) may be performed if assessment of mediastinum, hila, pleural space or chest wall is required (tumour staging, soft tissue infection etc.)

In patients in whom the differential diagnosis includes pulmonary embolism (dyspnoea, pleuritic chest pain etc.) intravenous contrast injection with a delay appropriate for CT pulmonary angiography may be required.

### Dose considerations

As many cancer patients are treated with a curative intent and these patients usually undergo several imaging studies involving radiation exposure the potential for dose reduction needs to be considered in every individual study, particularly at CT.

In addition to standard approaches such as iterative reconstruction tailoring of the radiation dose to the individual clinical situation is mandatory:

For example, in a patient with severe dyspnoea, in whom the differential diagnosis includes pulmonary infection, toxicity, and pulmonary embolism a contrast enhanced protocol with reduced kilovoltage (e.g. 80 kV) may be considered.

Unenhanced CT can usually be performed with significantly reduced tube current (20–40 mAs). The limitation of low-dose CT in detection of subtle density differences particularly ground glass may be overcome by using a narrower lung window setting than usually (window width 1000 Hounsfield Units (HU) instead of 1500 HU).

### Image acquisition and reconstruction

The technique of choice is multidetector helical CT, if possible during suspended inspiration.

Image reconstruction should be performed in the axial plane with a slice thickness of 3 - 5 mm in lung and soft tissue reconstruction algorithms.

Axial images at the minimal available slice thickness (e.g. 1 mm) should be reconstructed in the high-resolution kernel to allow for maximum spatial resolution and high-quality multiplanar coronal and sagittal reformations.

In our institution routine reporting is done on the basis of 5 sets of images, namely axial 3–5 mm images at soft tissue and lung windows, thin-slice mm axial images and coronal and sagittal reformations at lung window (Table 2).

**Table 2:** Standard image reconstruction and display

Axial plane	3–5- mm standard reconstruction kernel (soft tissues)
	3–5- mm high-resolution reconstruction kernel (lung)
	1–2 mm high-resolution reconstruction kernel (lung, multiplanar reformation)
Sagittal plane	1–5 mm high-resolution kernel
Coronal plane	1–5 mm high-resolution kernel

In the presence of a patient with significant dyspnoea and a normal chest CT in suspended inspiration, additional images obtained at end-expiration may be useful to detect small airways disease which may only present as air-trapping at expiratory images. Small airways disease may be due to infection or toxicity of oncologic drugs.

### Image display

On the reporting workstation images are routinely displayed with 4 images simultaneously:

- Axial 3–5 mm mediastinal window (400/20 HU: unenhanced, 400/40 HU enhanced)
- Axial 3–5 mm lung window (1500/- 600 HU)
- Coronal 3–5 mm lung window
- Sagittal 3–5 mm lung window

For analysis of the lung morphology, particularly in nodular, reticular and linear patterns, 1-2 mm axial (occasionally sagittal and coronal) images at lung windows are viewed.

Rarely maximum intensity projection images are reconstructed to better delineate micronodular pattern.

### Interpretation

#### Adequacy of the study

As patients with pulmonary disease even if correctly instructed may not be able to hold their breath at end-inspiration, the degree of inspiration is the first aspect of the analysis.

A reliable sign of a good inspiratory effort is an ovoid (convex) shape of the thoracic trachea. During expiration the posterior membranous part of the trachea (pars membranacea) is flat or even concave.

In cases with poor inspiration apparent ground glass opacity of the lung may be only due to expiratory effects. In these cases “ground glass” is usually observed in the posterior aspects of pulmonary lobes with a ventrodorsal gradient of density and may not be mistaken for true pathology. In these cases it may be appropriate to try and repeat the study with a better inspiratory effort.

### Analysis of radiological patterns

#### Morphology

Diffuse pathology of the lung demonstrated at CT is usually classified in 4 categories. This approach is also useful in suspected pulmonary complications of oncologic therapy.

### Consolidation/ground glass

In diffuse pulmonary infiltration there are two different morphologic types: a density that obscures pulmonary vessels and bronchial walls at lung window settings is termed “consolidation” whereas a less pronounced degree of increased density which does not obscure pulmonary vessels and bronchial walls is termed “ground glass”.

In consolidation the air in the peripheral airways bronchioles and alveoli is completely displaced by a solid substance such as pus, haemorrhage, tumour cells etc., whereas ground glass density is due to partial replacement of air independent of the type of substance.

### Nodular pattern

If a diffuse nodular pattern is present differentiation is made between three main types of pulmonary nodules depending on the exact location of the nodules with respect to the secondary pulmonary nodule, the smallest anatomical subunit of the lung:

- **Centrilobular (airspace) nodules** are usually ill-defined and are located in the centre of the secondary pulmonary nodules and are, thus, clearly separate from the costal pleura.
- **Perilymphatic nodules** are usually well-defined and are located along the intralobular septae and the visceral pleura and, thus, the interlobar fissures as well along the bronchovascular bundles.
- **Nodules with random distribution** are usually well-defined and are located in all parts of the lung with respect to the secondary lobule.

### Linear, reticular pattern

A linear or reticular pattern is usually caused by substrate accumulating in the inter- and intralobular septae. The finding is not specific as the substrate may represent fluid (interstitial oedema), inflammatory exudate (interstitial pneumonia) or tumor (lymphangitic carcinomatosis: LAC).

The distribution may help to distinguish oedema which is usually symmetrical with a dorsal and basal predominance from interstitial pneumonia and LAC which are usually asymmetrical or even unilateral.

The time course at follow-up may also help in the differentiation: Interstitial pattern in oedema may change rapidly (<24 h), in infection changes are less rapid (days) whereas in LAC findings remain relatively constant for days or weeks.

### Decreased attenuation

This feature summarizes emphysema, pulmonary cysts and air-trapping. Emphysema and pulmonary cysts do not usually represent complications of oncologic therapy. Bronchiolitis presenting as air-trapping (see above: expiratory CT) may be due to viral and bacterial infection or drug toxicity (e.g. methotrexate). Some mild degree of air-trapping is also observed in normal lung and other pulmonary diseases.

### Distribution

The distribution of pathological findings within the lung may be useful to differentiate between different conditions.

Apical versus basal predominance as well as peripheral versus central localisation are helpful in distinguishing between different diseases with similar morphological changes.

The fact, that abnormalities may cross anatomical borders and at the same time are confined to a known or presumptive radiation port is almost pathognomonic of radiation pneumonitis.

### Analysis of an individual case

As a first step the degree of inspiration needs to be assessed by checking whether the **trachea** appears **ovoid** with a convex pars membranacea indicating an appropriate degree of inspiration or whether the posterior aspect is flat or even concave indicating an expiratory scan. If so, apparent ground-glass density may have to be discarded, particularly when observed in the posterior portions of each pulmonary lobe (see above).

Secondly, it needs to be checked which of the morphological patterns of lung pathology is the predominant type as in most pathologic conditions a mixture of different patterns is observed.

### Consolidation/ground glass

In increased diffuse attenuation manifesting either as ground glass opacity or consolidation (see above) additional aspects may help to suggest the aetiology.

**Predominant consolidation with some ground glass and air space (centrilobular) nodules** suggests bacterial pneumonia.

A mixture of **consolidation and ground glass in combination with nodular and/or reticular pattern** is commonly observed in viral pneumonia.

**Focal areas of consolidation surrounded by a “halo” of ground glass** is the typical finding in angioinvasive aspergillosis in the appropriate clinical setting (severe neutropenia and fever that does not respond the antibacterial therapy).

**Ground glass or consolidation in an area of the lung that has been exposed to radiation therapy** with the appropriate dose and time interval is suggestive of radiation pneumonitis

**Predominant or pure ground glass with a diffuse, bilateral and central location** is typical of pneumocystis jirovecii pneumonia (PCP).

**Predominant ground glass with an upper lobe predominance** suggests acute hypersensitivity pneumonitis as one manifestation of drug-induced lung disease. The subacute stage is characterized by a **combination of ground glass and air-space nodules**.

### Linear or reticular pattern

**Bilateral symmetrical linear and reticular pattern**, usually with some ground glass and a predominance in the dependend portion of the lung is suggestive of interstitial oedema. It is usually associated with extrapulmonary findings of right heart failure such as bilateral pleural effusions (usually right > left), ascites, distension of SVC and IVC and gall bladder wall oedema.

The combination of **linear/reticular pattern with ground glass, consolidation and/or nodular pattern** is typical of viral pneumonia.

**Linear/reticular pattern often with a nodular appearance of the thickened septae** with an asymmetric or unilateral distribution with or without thickening of the bronchovascular bundles is found in lymphangitic spread of tumour.

A predominant **reticular pattern with a basal and peripheral distribution** in combination with ground glass is found in drug-induced lung disease with a NSIP pattern (non specific interstitial pneumonia).

### Nodular pattern

**Centrilobular nodules** are either due to inflammation (bronchiolitis/vasculitis) or spread of tumour along the alveolar septae (adenocarcinoma with predominant lepidic growth (formerly: bronchioloalveolar carcionoma).

Bronchiolitis may be due to infection (viral, bacterial, tuberculosis bronchiolitis) or may be drug induced, e. g. methotrexate). Vasculitis in this setting may be drug induced.

**Random pattern** of nodular disease is usually due to haematogenous spread of infection (candida, tuberculosis) or tumour (pulmonary metastases). Whereas the nodules in infection tend to have a similar size, random distribution of pulmonary nodules with different sizes suggest metastases. Nodule growth as demonstrated at follow-up or by review of a previous CT examinations is highly suggestive of malignancy (metastases) whereas new nodules can represent both metastases and infection.

**Perilymphatic nodular** pattern is found in lymphangitic carcinomatosis or unrelated diseases such as sarcoidosis or silicosis/coal worker’s pneumoconiosis. This pattern may also be due to benign intrapulmonary lymph nodes.

In lymphangitic carcinomatosis there is usually a combination with linear/reticular pattern and thickening of bronchovascular bundles. The distribution is mostly asymmetrical or even unilateral. Lymphadenopathy and (often unilateral) pleural effusion are common.

In sarcoidosis a combination with linear/reticular pattern and thickening of bronchovascular bundles in a symmetric distribution with central predominance is common. Bilateral hilar as well as mediastinal lymphadenopathy is also common, whereas pleural effusion is very uncommon in sarcoidosis.

In silicosis the nodular pattern is found in an upper lobe and central predominance, association with hilar and mediastinal lymphadenopathy (with longer duration with peripheral calcification (eggshell lymph nodes) is very common. Pleural effusion is uncommon.

**Several larger nodules**, often with cavitation and ill-defined margins are typically found in septic emboli from bacterial infection in the venous system (central line, port-a-cath, septic thrombosis) or the right heart/endocarditis).

### Extrapulmonary findings

#### Pleural effusion

Bilateral pleural effusion is a common presentation of (right) cardiac failure, renal failure or hyperhydration which may be therapy-induced (cardiotoxic drugs, nephrotoxic drugs, large amounts of fluid during chemotherapy). In this setting the amount of pleural effusion is usually larger in the right than in the left pleural space.

There is an association with other signs of right heart failure such as pericardial effusion, ascites, dilatation of SVC, IVC and hepatic veins and gallbladder wall oedema.

Unilateral pleura effusion is usually not due to the courses listed above but either pleural carcinomatosis or pleural infection. Diffuse pleural thickening with or without contrast enhancement (“split pleura sign”) may be found in infection, chronic non-infected effusion or pleura carcinomatosis. Nodular pleura thickening is almost pathognomonic of pleural tumour spread (carcinomatosis, lymphomatosis, sarcomatosis). This may be associated with lymphangitic carcinomatosis and lymphadenopathy.

### Lymphadenopathy

**Mildly enlarged lymph nodes** are usually found in heart failure but also in drug-induced lung disease, radiation pneumonitis and many other unrelated conditions.

**More pronounced lymphadenopathy** is found in bacterial and viral pneumonia with the location of enlarged lymph nodes according to the lymphatic drainage of the infected pulmonary lobe or segment.

The **most pronounced lymph node enlargement** if found in malignant lymphadenopathy. Lymph nodes with a diameter > 3 cm are usually malignant (malignant lymphoma, lymph node metastases).

**Lymphadenopathy with rim-enhancement** is typical of tuberculous lymphadenitis which may occur without pulmonary manifestations of tuberculosis.

Lymphadenopathy is uncommon in pneumocystis jirovecii pneumonia (PcP) or fungal infection (candida, aspergillus).

Lymphadenopathy due to unrelated diseases such as collagen vascular disease etc. is usually mild.

### Tumour manifestations

Extrapulmonary tumour manifestations need to be observed when interpreting pulmonary findings. Although possible it is unlikely that new or progressive pulmonary pathology presents malignant disease such as (LAC or metastases) when other tumour manifestations (axillary lymph node metastases, liver metastases, skeletal metastases) respond to therapy.

### Formulating the report

The report should, obviously, include the clinical question so as to to address it in the conclusion.

The examination technique should be described, particularly whether the CT examination was performed with or without intravenous contrast injection.

The quality of the examination (inspiration, breathing artefacts) should be mentioned to show potential limitations of the study.

The morphology of pulmonary and extrapulmonary pathology should be described to allow re-evaluation if new aspects of the clinical context are identified.

Incidental findings that are not known are uncommon when cancer patients are imaged for potential complications of therapy as previous imaging (staging examinations) are usually available.

## Conclusion

In the setting of suspected complications of cancer therapy usually a **synopsis of clinical information** (signs and symptoms, body temperature), **history** (date and dose of radiation therapy, radiation portal, date and dose of systemic therapies, other relevant diseases such as DVT), **laboratory findings** (CRP, leucocytes, bronchoalveolar lavage (BAL) results: lymphocytic, neutrophil alveolitis, organisms, evidence of viral pneumonia) and **imaging findings** is made.

As a definite diagnosis can be rarely made, the degree of confidence in the diagnosis needs to be addressed.

### Recommendation

As there are few pathognomonic CT findings in the setting of suspected pulmonary complications of oncologic therapy recommendations on further procedures should be made in the report.

**Short-term follow-up** (days) may be useful to differentiate between oedema versus infection versus tumour progression as oedema may exhibit marked changes within 24 h with the appropriate therapy, infection usually requires days for improvement or deterioration of pulmonary abnormalities whereas tumour manifestations remain relatively stable for weeks.

In suspected drug toxicity **discontinuation of the potentially offending agent** with or without steroid therapy and usually parallel antibiotic therapy to cover potential infection may be advocated. Additional non-radiological diagnostics procedures such as bronchoalveolar lavage (BAL), transbronchial biopsy (TBB) or percutaneous biopsy may be recommended and guided by CT.

### Summary

In cancer patient during or following oncologic therapies with respiratory symptoms and pulmonary pathology at chest CT the differential diagnosis includes infection, therapy-induced disease and tumour progression.

Although CT morphology may be typical or even pathognomonic in some conditions the diagnosis is usually made by a synopsis of imaging, clinical and laboratory features.

Close communication with referring colleagues and a good knowledge of potential side effects of therapeutic concepts, their time course and CT morphology is crucial in the differential diagnosis.

## Appendix

### Important features in different complications of oncologic therapy

This list contains some – but certainly not all – features that may help in the diagnosis

### Bacterial pneumonia

- Asymmetrical/unilateral
- Consolidation, ground glass, air space nodules
- Pleural effusion
- lymphadenopathy

### Viral pneumonia

- Reticular or linear pattern
- Nodular pattern
- Ground glass
- Consolidation

### Fungal pneumonia

- Prolonged neutropenia, not responsive to antibacterial therapy
- Chest radiograph may be normal
- Invasive pulmonary aspergillosis: consolidation with halo of ground glass, infarct-löike pattern
- Candida. Multiple diffuse, bilateral, random nodules, ususally candidiasis of mouth, oesophagus, vagina etc.

### Pneumocystis jirovecii pneumonia (PCP)

- Severe dyspnoea/hypoxia
- Chest radiograph may be normal
- At CT bilateral, diffuse, central ground glass
- No pleural effusion
- No lymphadenopathy

### Oedema

- Diffuse, bilateral reticular pattern in the dependend portion of the lung
- Some ground glass
- Bilateral pleural effusion
- Mild lymphadenopathy

### Cryptogenic organizing pneumonia (COP)

- Focal areas of consolidation
- Not responsive to antibacterial therapy
- May be associated with radiation therapy
- May be a manifestation of drug-induced lung disease

### Radiation pneumonitis

- Ground glass or consolidation limited to the radiation port
- 6 weeks or later after threshold of 30–40 Gy to the involved lung has been reached
- Typical time course: ground glass - consolidation - fibrosis

### Drug-related lung disease

- Think of it !
- www.pneumotox.org
- may manifest with large variety of pulmonary patterns including NSIP, hypersensitivity pneumonitis, COP, oedema, haemorrhage, bronchiolitis, vasculitis, pleural effusion etc.
- diagnosis of exclusion
- therapy: cessation of offending drug +/- steroids

### Lymphangitic carcinomatosis (LAC)

- Linear and reticular pattern
- Nodular perilymphatic pattern
- Asymmetrical or unilateral
- +/- pleural effusion
- +/- lymphadenopathy
- +/- pulmonary metastases

## Abbreviations

BAL: Bronchoalveolar lavage
COP: Cryptogenic organizing pneumonia
CRP: C-reactive protein
DVT: Deep venous thrombosis
Gy: Gray
HU: Hounsfield Units
iMRT: Intensity-modulated radiation therapy
IPA: Invasive pulmonary aspergillosis
IVC: Inferior vena cava
kV: Kilovolt
LAC: Lymphangitic carcinomatosis
mAs: Milliampereseconds
NSCLC: Non small-cell lung cancer
NSIP: Non-specific interstitial pneumonia
PCP: Pneumocystis jirovecii pneumonia
PE: Pulmonary embolism
SVC: Superior vena cava
TBB: Transbronchial biopsy

## References

[1] Choi YW, Munden RF, Erasmus JJ, Park KJ, Chung WK, Jeon SC, Park CK (2004). Effects of radiation therapy on the lung: radiologic appearances and differential diagnosis. Radiographics.

[2] Mosvas B, Raffin TA, Epstein AH, Link CJ (1997). Pulmonary radiation injury. Chest.

[3] Souza FF, Smith A, Araujo C, Jagannathan J, Johnston C, O’Regan K, Shinagare A, Ramaiya N (2014). New targeted molecular therapies for cancer: radiological response in intrathoracic malignancies and cardiopulmonary toxicity: what the radiologist needs to know. Cancer Imaging.

[4] Crestani B, Kambouchner M, Soler P, Crequit J, Brauner M, Battesti JP, Valeyre D (1995). Migratory bronchiolitis obliterans organizing pneumonia after unilateral radiation therapy for breast carcinoma. Eur Resp J.

[5] Sousou T, Khorana A (2007). Inpatient chemotherapy and risk for venous thromboembolism. J Clin Oncol.

